# Automatic Assessment of Tone Quality in Violin Music Performance

**DOI:** 10.3389/fpsyg.2019.00334

**Published:** 2019-03-14

**Authors:** Sergio Giraldo, George Waddell, Ignasi Nou, Ariadna Ortega, Oscar Mayor, Alfonso Perez, Aaron Williamon, Rafael Ramirez

**Affiliations:** ^1^Music Technology Group, Music and Machine Learning Lab, Department of Communications and Technology, Pompeu Fabra University, Barcelona, Spain; ^2^Centre for Performance Science, Royal College of Music, London, United Kingdom; ^3^Faculty of Medicine, Imperial College London, London, United Kingdom

**Keywords:** automatic assessment of music, machine learning, violin performance, tone quality, music performance

## Abstract

The automatic assessment of music performance has become an area of increasing interest due to the growing number of technology-enhanced music learning systems. In most of these systems, the assessment of musical performance is based on pitch and onset accuracy, but very few pay attention to other important aspects of performance, such as sound quality or timbre. This is particularly true in violin education, where the quality of timbre plays a significant role in the assessment of musical performances. However, obtaining quantifiable criteria for the assessment of timbre quality is challenging, as it relies on consensus among the subjective interpretations of experts. We present an approach to assess the quality of timbre in violin performances using machine learning techniques. We collected audio recordings of several tone qualities and performed perceptual tests to find correlations among different timbre dimensions. We processed the audio recordings to extract acoustic features for training tone-quality models. Correlations among the extracted features were analyzed and feature information for discriminating different timbre qualities were investigated. A real-time feedback system designed for pedagogical use was implemented in which users can train their own timbre models to assess and receive feedback on their performances.

## 1. Introduction

In recent years, several computational systems have been developed with the aim of enhancing music education and instrument tuition. In these systems automatic assessment of musical performance plays a central role. However, human assessment is often subjective, thus making the implementation of an automatic assessment system a significant challenge. In music education, assessment relies on consensus of highly trained experts who produce subjective interpretations of performance (Thompson and Williamon, 2003; McPherson and Schubert, 2004). Even reducing musical performance to its simplest component part (i.e., a single tone) still poses a challenge (Zdzinski, 1991). From a technical perspective, the tone quality of a performed sound is a result of numerous acoustic properties including pitch, loudness, and harmonic content (Terasawa et al., 2005; Eerola et al., 2012; Elliott et al., 2013). In contrast, the language used by musicians to describe tone can be highly personal without clear correspondence with the psychoacoustic properties they describe and can be affected by changes in pitch and dynamics (Melara and Marks, 1990). In this paper we consider timbre and tone as the same attribute of sound.

Attempting to obtain reliable models for the assessment of music performance involve several challenges. On one hand, most of the computational systems for music education rely only on pitch and timing accuracy assessment, leaving aside other relevant aspects of musical interpretation, such as timbre quality. On the other hand, a high degree of subjectiveness exists regarding the definition of high-level semantic labels for tone quality among music experts, which complicates significantly the generation of timbre models consistent with the experts' semantic labels. This is particularly evident in instruments such as the violin, where the quality of tone is a particularly relevant aspect in the overall quality of a musical performance (Hodgson, 1934; Galamian, 1962).

In this paper we present a machine learning approach for the automatic assessment of tone quality in violin music performance. Our aim is firstly to study the correlations between expert-defined tone quality semantic labels found in the literature and the features extracted from the audio signal; secondly, to generate machine learning models to classify different tone quality dimensions of violin sounds based on audio features; and thirdly to incorporate the obtained models in a technology-enhanced violin learning system to provide real-time feedback of such tonal dimensions. We recorded audio examples of expert-defined tone qualities performed by a professional violinist and collected violin recordings and tone labels from the Good Sounds Dataset (Romani Picas et al., 2015). We performed perceptual tests using expert-defined tone labels and studied the perceptual correlations among the labels. We extracted high and low-level features from the audio recordings, including both global and frame based descriptors. We applied automatic feature selection methods and machine learning techniques to obtain tone quality computational models based on the selected descriptors. Finally, the obtained tone quality models were used to implement a real-time visual feedback system for tone quality assessment in which, in addition to the experts tone labels, users are able to train their own tone models by recording examples of their own tone quality labels and obtain real-time visual feedback on the quality of those tone labels. The fact that the system allows tone quality labels to be defined by the users is a key aspect of the system aiming to address the problem arising from the subjectivity of the terms used by musicians to describe timbre in music.

## 2. Systems for Automatic Assessment of Music Performance

### 2.1. Automatic Accompaniment Systems and Score Followers

Most of the systems for automatic music performance assessment are based on audio signal processing technologies widely used in music information retrieval (Dittmar et al., 2012). However, while not explicitly providing assessment information or grading, some systems simply provide automatic accompaniment to enrich the practicing and performance of soloist music. Such is the case of Music Plus One (Raphael, 2010), a system for musical accompaniment in which orchestral music follows the soloist timing variations by means of a Hidden Markov Model. Antescofo (Cont, 2008) is a score following system which allows the recognition of the player position and tempo in a score. It can be used in soloist-accompaniment scenarios as well as a compositional tool in which electronic events can be triggered from events in the soloist performance.

### 2.2. Systems for Automatic Assessment Based on Pitch and Onset Detection

Most of the systems that provide assessment of a performed musical piece are based on pitch and onset accuracy assessment. Pitch and onset detection are two low-level music information retrieval audio descriptors for which a number of algorithms and methods are publicly available in different programming languages and libraries. Song2see (Cano et al., 2011) is a gaming software for music learning and practicing. It makes use of pitch detection and source separation to allow a user to play music with traditional instruments (guitar, bass piano, saxophone, flute, and voice). The system uses its own score rendering format in order to provide a visualization of the current time position over the score. The system returns a score based on the correctness of performed notes. Other commercial systems such as Yousician^1^ and Smart Music^2^ are able to provide real-time feedback of music performance. SmartMusic is developed by MakeMusic, which provides tools to practice band and orchestral music parts. Among features to enhance student/teacher remote interaction and tuition follow up and feedback, the system is able to provide real-time feedback on pitch and timing accuracy providing a score after recorded takes.

### 2.3. Systems for Automatic Characterization of Music Performance

Several systems have been developed aimed at characterizing other musical performance aspects beyond pitch and onset detection, such as timbre or articulation. Even though these systems do not aim to provide an explicit score/grading of the performance, the information retrieved by this type of system may be used for that purpose. In the context of expressive music performance, the automatic characterization of dynamics and articulation from low-level audio features has been studied (Maestre and Gómez, 2005). In Percival (2013), Support Vector Machine (SVM) models are trained to evaluate a violin synthesizer. However, the aim of the system is not automatic evaluation of real violin sounds but to fit the bowing parameters (e.g., force) in the physical model synthesizer by incorporating a training loop of the system with the inputs given by a listener.

Other approaches emphasize the automatic assessment of tone quality in trumpet sounds using machine learning techniques (Knight et al., 2011). Good-Sounds (Romani Picas et al., 2015) makes use of machine learning techniques to identify good and poor quality notes in trumpet, clarinet and flute performance. The modeling strategy is based on training data consisting of low and high-level audio features, extracted from recorded good and bad musical note examples. Giraldo et al. (2017a,b) proposed a system to assess automatically the quality of timbre in violin sounds using machine learning techniques.

### 2.4. Characterization of Tone

Several studies have attempted to characterize timbre (tone) quality and its implications on the quality of music performance. Saitis et al. (2017) studied how musicians conceptualize aspects of sound quality and performance in violin music by means of interviews with performers, where associations among perceptual evaluations and physical description were addressed. The relation of the dynamic behavior of the violin and perceived quality has been investigated by several studies trying to identify such verbal attributes. Dünnwald (1991) suggests four quality regions in the violin based on its frequency response. Similar studies characterize frequency ranges for violin tone and projection (Hutchins, 1989), as well as ranges for tonal attributes such as soft/harsh and dark/bright (Schleske, 2002). Violin sound projection was studied by Loos (1995) in terms of the perceived nearness of sound. Štěpánek and Otčenášek (1999) reported on associations among several tone qualities such as sharp/narrow with high/low spectral centroid values and rustle with temporal energy changes. Similarly, Lukasik (2005) suggested associations among spectral centroid with bright/dark, and tristimulus 1 and 3 with deep/full and flat/empty, respectively. Saitis et al. (2015) reported on associations among spectral centroid, tristimulus 2 and 3 with sound richness. Hermes et al. (2016) reported high correlations among harmonic centroid and clarity.

Several studies have investigated the verbal description and/or components of tone and timbre quality. Research aiming to obtain representative timbre spaces have been conducted in the past by means of perceptual similarity experiments (Grey, 1977; Grey and Gordon, 1978; Iverson and Krumhansl, 1993; McAdams et al., 1995; Lakatos, 2000). Studies aiming to find semantic labels for characterizing timbre and its acoustical correlates have been performed by searching adjectives used consistently to describe acoustical tonal features, as well by performing surveys on the verbalization of the description of several timbre stimuli (Moravec and Štepánek, 2003; Nykänen and Johansson, 2003; Lukasik, 2005; Disley et al., 2006; Sarkar et al., 2007).

### 2.5. Signal Processing Perspectives

From the computational perspective timbre has been studied in terms of its relation to the audio descriptors that can be mathematically computed from the digital audio signal. In general, machine learning techniques are used to find patterns that permit the recognition of different timbre qualities from the descriptors extracted from the audio signal (De Poli et al., 1993; Toiviainen et al., 1995; De Poli and Prandoni, 1997; Loureiro et al., 2004). Alluri and Toiviainen (2010) devised subjective rating scales to quantify perceptual qualities of timbre to correlate them later with features extracted from the audio signal. Knight et al. (2011) studied tone quality in brass instrument performance based on subjective ratings of good/bad timbre among sounds with the same pitch and loudness played by the same instrument. Support Vector Machines (SVM) were used to discriminate good and bad sounds based on different score thresholds and groupings. Romani Picas et al. (2015) studied overall goodness of flute, clarinet, and trumpet sounds. The quality of a performed sound was defined based on its dynamic pitch and timbre stability, timbre richness, and attack clarity. Based on recordings of good and bad examples of each of the aforementioned sound qualities, machine learning models were obtained to classify performed sounds in real-time.

## 3. Materials and Methods

**Figure 1:**
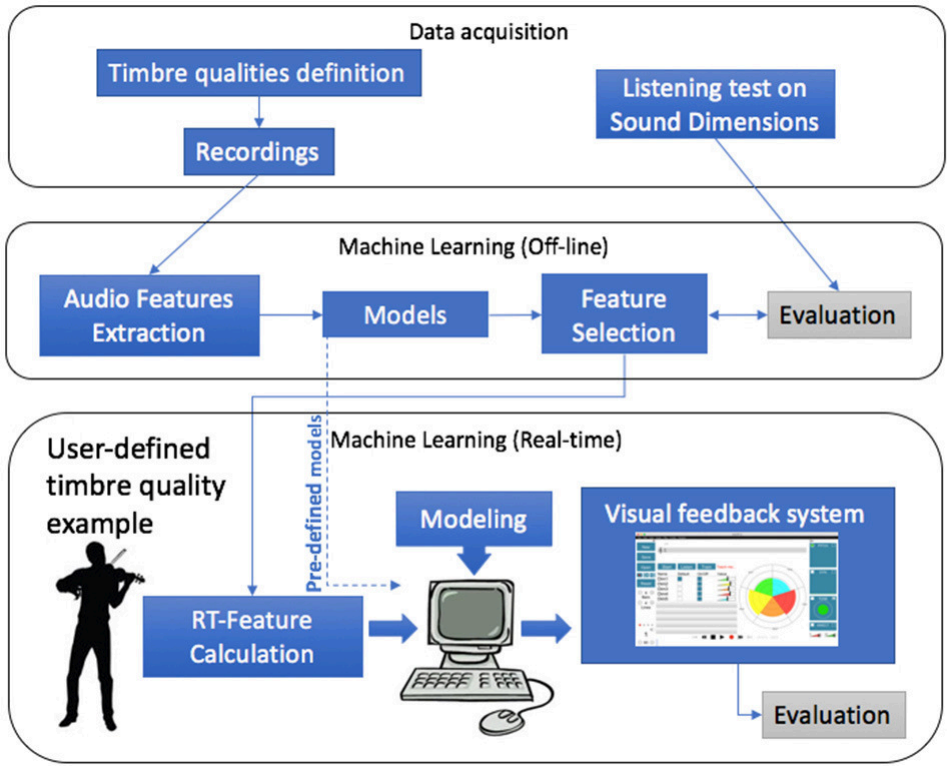
Overall framework for automatic tone assessment using machine learning.

The methodology used in this study is depicted in Figure 1 and can be subdivided into three main blocks: data acquisition, offline machine learning modeling, and user-defined machine learning modeling. First, we obtained definitions of tone qualities from music experts and recorded examples of each of them. Second, we collected data on the perception of the defined qualities from listeners. Additionally, we made use of machine learning techniques to obtain models to predict the tone quality dimensions from recorded sounds. The obtained models were later used in the system as pre-defined models. Using automatic feature selection tools we obtained a subset of features that best predicted the tonal qualities. Finally, we used the obtained set of features to perform a user-defined machine learning modeling approach, in which a user can train tone quality models with his/her own set of tonal quality sound examples to obtain visual feedback on the assessment on the quality of new performed sounds.

### 3.1. Data Acquisition

#### 3.1.1. Semantic Definition of Tone Qualities

Tone qualities for evaluation were chosen using the semantic differential method, in which each tone is measured against bipolar scales with opposing extremes (e.g., loud-soft; Zacharakis et al., 2014, 2015). While alternative methods employing a magnitude estimation of each individual item have been employed with some success (e.g., Kendall and Carterette, 1993), an opposing semantic differentials approach was chosen to reduce the number of evaluations required by the participants and following discussion with expert violinists of their use in pedagogical practice. A bipolar conceptualization of tonal space has been used in previous studies (e.g., Lichte, 1941; von Bismarck, 1974a,b; Pratt and Doak, 1976; Moravec and Štepánek, 2003) highlighting in particular the features of brightness, roughness, and fullness. Such studies have often employed artificially-generated tones or made cross-instrument comparisons; while the present research examined the perception of violin tones, an idiosyncratic list of 10 opposing semantic pairs was created through discussion with English-speaking expert violinists and a review of the existing literature (see Table 1). A total of 20 tonal dimensions, grouped in 10 opposite pairs, were considered and are presented in Table 1.

**Table 1:** Tonal semantic dimensions defined by music experts.

Dark	Bright
Cold	Warm
Harsh	Sweet
Dry	Resonant
Light	Heavy
Grainy	Pure
Coarse	Smooth
Closed	Open
Restricted	Free
Narrow	Broad

#### 3.1.2. Recorded Material

The recorded material used in this study consisted of recorded examples of violin tones. Two sets of recordings were considered: first, the publicly available data set of recorded sounds from the Good-Sounds project (Romani Picas et al., 2015), which included a set of recorded violin sounds with good and bad examples of five of the sound quality dimensions (see section 2.5). This data set was initially used for machine learning modeling in the preliminary studies presented by Giraldo et al. (2017a,b) and as baseline for the perceptual tests. Second, we obtained recorded examples of the aforementioned expert-defined tonal semantic dimensions (see section 3.1.1) from a professional violinist^3^. The examples were recorded using fingered notes on each of the four strings, in both first and fifth position, using each finger (one to four), and repeating each note using up/down bow strokes. Sixteen notes per tonal dimension were recorded for a total of 320 notes.

#### 3.1.3. Perceptual Tests

An online survey on the perception of the tonal qualities considered was performed following a similar approach used by Alluri and Toiviainen (2010). On one hand, our aim was to investigate the semantic associations of listeners and the defined tonal dimensions based on measurements of inter-user and inter-dimension correlations. On the other, we were interested in comparing the performance of the models with the discriminative perceptual abilities of human listeners by obtaining a confusion matrix of how accurate each tonal dimension was differentiated. Finally, we were interested in investigating the correlations among the expert-defined tone quality dimensions and the scales for goodness defined by Romani Picas et al. (2015).

The survey was implemented using the Flask framework, where data were collected over an SQL server platform, in an online web-based questionnaire. A total of 22 sound samples were used where the violinist consecutively performed four different notes, repeating each one using up and down strokes (one on each string/finger) across the violin tesitura (see section 3.1.2), always using the same four notes for each of the defined tonal quality dimensions.

*Participants:* There were 20 respondents (28% female) with a mean age of 36.49 years (*SD* = 5.71, range = 29–51). They had a mean musical experience of 11.8 years (*SD* = 7.9, range = 1–25 years), with representation from professional (23%), student (19%), and amateur (55%) groups and 100% having taken formal lessons on their primary instrument for a mean 5.15 years (*SD* = 3.6, range = 1–10). The cohort was represented 8 nationalities, with a significant proportion being Spanish (63%). The range of primary instruments included violin (36%) and guitar (35%) with the remaining (29%) comprising a mix of percussion, vocal, and other instruments. One third (34%) of the cohort reported classical as their primary genre, with the remaining comprising jazz, folk, pop, and other. The survey opened with an information sheet outlining the topic and purpose of the study and instructing respondents that, by beginning the survey, they were providing informed consent. Ethical approval for the study, including consenting procedures, was granted by the Conservatoires UK Research Ethics Committee following the guidelines of the British Psychological Society.

*Procedure:* Respondents were prompted with an initial page with written instructions followed by a form to collect demographic information and musical background information. A form was then presented containing a sound player with the corresponding sound sample and a list of the tone quality dimensions (see Table 1) as well as the semantic tonal dimensions defined by Romani Picas et al. (2015), in which a grid of radio buttons were presented in between each bipolar opposite where the participant could provide a score. The sound player permitted the listeners to play the audio excerpt as many times as they wished. After listening to a sound example, participants were instructed to rate each of the expert-defined tone quality dimensions on a seven-point Likert scale. The sound samples were randomly presented to each user. Two randomly chosen sound examples were selected to be repeated during the test in order to assess user consistency.

### 3.2. Machine Learning Modeling: Offline Approach

This stage of the study was carried out initially in an offline setting. Initial preprocessing of the data was carried out by eliminating outliers and extreme values (which might be produced by peaks or artifacts in the audio signal and/or errors in pitch/harmonic peak detection). Data filtering was performed using an interquartile range (IQR) filter with an extreme value factor set to three times the IQR and an outlier value factor of 1.5 times the IQR.

#### 3.2.1. Feature Extraction

Audio descriptors help to characterize an excerpt of an audio signal (or an audio frame) in terms of its spectral, temporal, and spectro-temporal properties. Audio descriptors are divided into global and instantaneous descriptors. The former refer to those in which the whole signal is used for its computation (e.g., attack duration), the latter are computed at a frame-level for each frame at a time. For each of the audio excerpts, we extracted frame-based low-level audio descriptors (see Peeters, 2004 for an overview) using the Essentia library (Bogdanov et al., 2013). Low-level audio descriptors included pitch, spectral (e.g., spectral centroid, spectral kurtosis, MFCCs, etc.), and energy descriptors (e.g., RMS). A total set of 95 audio features were considered, from which 35 were frame-based and the remaining were global.

#### 3.2.2. Feature Selection

Only spectral frame-based low level descriptors were considered given the nature of the implementation, taking into consideration the review of the state-of-the-art in timbre quality and audio description associations (see section 2). On one hand, real-time feedback requires fast descriptor computation and thus frame based descriptors are a natural choice. On the other, spectral descriptors are obtained from the information contained in the spectrogram of the audio wave, which has a direct relation with timbre. Global descriptors were considered as well by computing the mean and the standard deviation of the frame-based descriptors over a sliding window of 600 ms. The computation of these aim to encode information on the stability of the studied timbre qualities of a performed note over time.

Automatic feature selection using filter methods was used to obtain the subset of features most relevant for classification. Filter methods use a proxy measure (e.g., information gain) to score features, where these are filtered and ranked by information gain values. We used the methods provided by the Weka library (Hall et al., 2009). The list of the descriptors considered for this study with corresponding descriptions is presented in Table 2.

**Table 2:** List of audio features.

**Feature**	**Info gain**
Pitch	Fundamental frequency in Hz
Energy	Mean Square Root over a 600 ms window
Tristimulus1	Relation of the first fundamental harmonic over the total of harmonic peaks
Tristimulus2	Relation of the second plus the third harmonic peak over the total of harmonic peaks
Tristimulus3	Relation of the remaining harmonic peaks after the third over the total of harmonic peaks
specCent	The spectral center of gravity of the spectrum
specSpread	The spectral standard deviation
specSkew	Measure of the asymmetry of the spectrum around its mean value
specKurt	Measure of the flatness of the spectrum around its mean value
specSlope	Computed from the slope of the linear regression over the spectral amplitude values
specDecr	Averages the set of slopes of the lowest frequencies
specRolloff	Defined as the frequency below which 95% of the signal energy is contained
specFlat	Ratio between the geometric and the arithmetic mean of the spectrum
specCrest	Ratio between the maximum arithmetic mean and the arithmetic mean of the spectrum
MFCC	Mel frequency cepstral coefficients

#### 3.2.3. Modeling

After extracting audio descriptors from the recorded audio samples, several machine learning models were trained and compared. The machine learning algorithms taken into consideration were: Linear Regression, Support Vector Machines (SVM) with radial kernel, and Artificial Neural Networks (ANN) with one hidden layer (half the size of the input nodes). Offline tests were performed using the Weka machine learning library (Hall et al., 2009). In particular, for SVM we applied the Sequential Minimal Optimization (SMO) algorithm for training a support vector classifier, which uses a “one vs. all” approach for multi-class classification problems.

Classification experiments were carried out over the expert-defined tone qualities subset. Our aims were to obtain classification models for the defined paired labels for tone quality and to obtain a subset of the audio features that best predicted each of the aforementioned tonal qualities. We considered two main approaches for the modeling process:

1. Multi-class classification to predict each of the 20 tone labels.
2. Binary classification to obtain models to classify contrary pairs of the expert-defined labels (e.g., dark-bright, cold-warm, etc.).

Several sub-groupings were considered to test the consistency of the obtained models across several scenarios as follows:

1. By pitch range: The aforementioned modeling strategies were carried out on instances grouped by pitch range. We considered a distribution of low, medium, and high registers. This subdivision was done by octaves over the violin register, i.e., the first octave (from G2 to G3) was considered “low,” second octave (from G3 to G4) was considered “medium,” and notes above G4 were considered “high” register.
2. By position: The modeling strategies took into consideration whether notes were played in the 1st or 5th position of the violin. This distribution resulted from how the recordings were played by the violinist. (Other positions could have been considered, but these will be left as a possible extension for future work.)
3. By finger: The modeling strategies were used in subsets defined by the finger used to play a particular note.

### 3.3. Machine Learning: User-Defined Approach

The subjective nature of timbre/tone perception and label semantics produces several complications for obtaining a predictive model to generalize to different performers and different performance conditions (e.g., instrument qualities, acoustic conditions of the room, quality of the audio capture hardware, etc.). As a result, the motivation of a user-defined machine learning approach was to offer a tool able to classify audio samples in real-time based on training examples given by a user, where semantic labels of tone quality can be user-customized.

#### 3.3.1. Real-Time Feature Computation

Based on the results of feature selection from the offline approach (see section 3.2.2), for this approach we considered the features presented in Table 2. Features were computed on a frame basis in real-time, where global descriptors (i.e., mean and standard deviation) were computed on a 600 ms historic sliding window.

#### 3.3.2. Modeling

For the user-defined modeling approach we used the same three machine learning methods mentioned in section 3.2, where the ANN was set as default based on the offline machine learning analysis (see section 4.3). The system permits the storage of the recorded data of the computed features along with their respective user-defined labels as well as the generated models. A detailed explanation on the real-time implementation is presented in the results section 4.4.

## 4. Results

### 4.1. Tone Survey

Consistency among participants' ratings was assessed using Cronbach's coefficient (alpha) and is presented in Table 3. An acceptable degree of reliability was obtained (alpha > 0.80; Mcgraw and Wong, 1996) for all the sound examples. Table 4 shows the mean correlation among dimensions (i.e., inter-dimension correlation). Similarly, Table 5 shows the correlations among the proposed tonal qualities and the ones used by Romani Picas et al. (2015). Higher correlations (i.e., CC > 0.8) were obtained between grainy/pure with coarse/smooth, and restricted/free with narrow/broad, which indicated that these groups of tonal dimensions could be perceived as having the same perceptual quality. Average correlations between 0.6 and 0.7 among similar labels was also found for closed/open, restricted/free, and narrow/broad.

**Table 3:** Inter-subject correlations and Cronbach's alpha for the tone quality dimensions perceptual study.

**Recorded tone quality**	**Mean inter-subject r**	**Cronbach's alpha**
Bright	0.25	0.7
Dark	0.29	0.7
Cold	0.30	0.7
Warm	0.27	0.8
Harsh	0.28	0.6
Sweet	0.25	0.8
Dry	0.28	0.8
Resonant	0.28	0.8
Light	0.29	0.7
Heavy	0.31	0.8
Grainy	0.27	0.9
Pure	0.29	0.8
Coarse	0.34	0.8
Smooth	0.27	0.6
Open	0.24	0.9
Closed	0.25	0.9
Restricted	0.24	0.9
Free	0.27	0.9
Narrow	0.35	0.9
Broad	0.27	0.8

**Table 4:** Inter-dimension correlations for the tone quality dimensions perceptual study.

	**Dark/bright**	**Cold/warm**	**Harsh/sweet**	**Dry/resonant**	**Light/heavy**	**Grainy/pure**	**Coarse/smooth**	**Closed/open**	**Restricted/free**	**Narrow/broad**
Dark/bright	1.00									
Cold/warm	−0.12	1.00								
Harsh/sweet	0.28	0.50	1.00							
Dry/resonant	0.14	0.41	0.70	1.00						
Light/heavy	−0.48	0.08	−0.35	−0.26	1.00					
Grainy/pure	0.34	0.30	0.79	0.55	−0.38	1.00				
Coarse/smooth	0.26	0.21	0.68	0.47	−0.36	0.87	1.00			
Closed/open	0.37	0.29	0.62	0.59	−0.25	0.62	0.51	1.00		
Restricted/free	0.39	0.26	0.63	0.55	−0.23	0.69	0.67	0.67	1.00	
Narrow/broad	0.29	0.32	0.68	0.60	−0.13	0.66	0.63	0.68	0.81	1.00

**Table 5:** Inter-dimension correlations for expert-defined tone quality dimensions vs. Good Sounds scales.

	**Overall/rating**	**Pitch/stability**	**Timbre/stability**	**Dynamic/stability**	**Attack/clarity**	**Timbre/richness**
Dark/bright	0.16	0.13	0.17	0.10	0.17	0.19
Cold/warm	0.32	0.22	0.19	0.18	0.33	0.37
Harsh/sweet	0.71	0.49	0.64	0.39	0.60	0.70
Dry/resonant	0.51	0.39	0.43	0.21	0.44	0.49
Light/heavy	−0.24	−0.22	−0.28	−0.14	−0.13	−0.18
Grainy/pure	0.75	0.52	0.72	0.49	0.57	0.71
Coarse/smooth	0.75	0.53	0.71	0.52	0.47	0.74
Closed/open	0.52	0.44	0.49	0.27	0.56	0.53
Restricted/free	0.58	0.47	0.55	0.41	0.43	0.66
Narrow/broad	0.58	0.44	0.56	0.38	0.52	0.70

**Figure 2:**
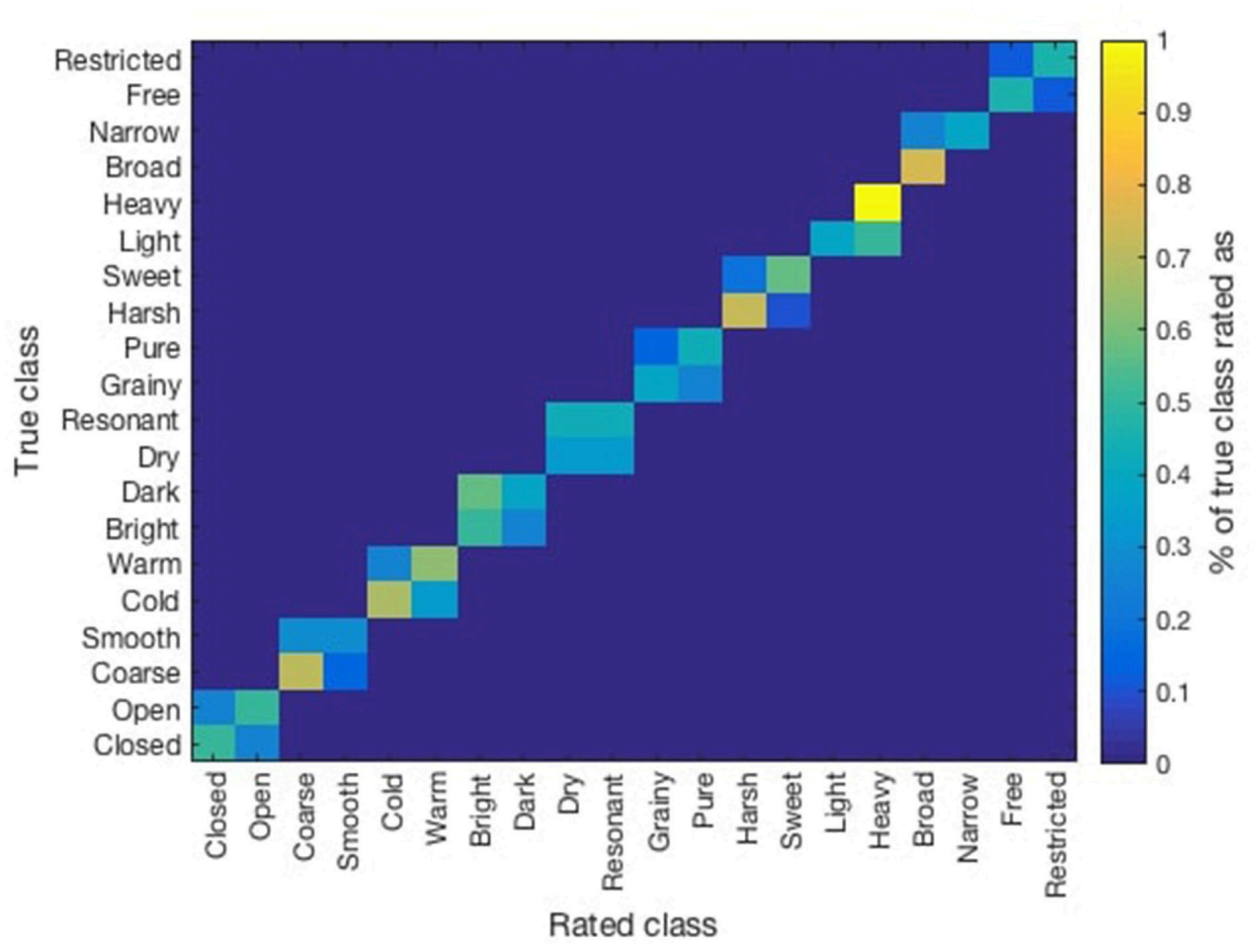
Confusion matrix of the obtained ratings of the listener over the considered recorded tonal qualities.

Previous results showed high correlations among similar opposite scales: e.g., narrow/broad and restricted/free (see Table 4). Similarly, low inter-subject correlation was found among listeners. This might have been a consequence of the rating system used, where each sound (recorded with one tonal attribute) was rated in all 10 opposite scales by listeners. Therefore, we compared the ratings obtained for each sound on its particular tonal quality. In Figure 2 we present a confusion matrix which was obtained by averaging the ratings (normalized from 0 to 1) obtained for each sound on its corresponding tonal quality. Higher values over the diagonals of each set of squares indicate that listeners rated correctly the intended tonal quality on the recording (e.g., light/heavy), whereas squares with more homogeneous values indicate listeners were not able to discriminate the intended tonal quality (e.g., dry/resonant).

Listeners reported having perceived that some of the adjectives (dark/bright, cold/warm, dry/resonant, closed/open, light/heavy, grainy/pure) correlated with the quality of violin/microphone used for recordings. A future approach would obtain recordings on the same set of tone qualities by different performers/instruments. Listeners also indicated that other adjectives such as restricted/free and narrow/broad were perceived with relation to the dynamics of the performance. Similarly, some mentioned that dimensions such as broad/narrow and open/close were related to the level of vibrato. Closer study of these aspects will be regarded in future work.

### 4.2. Feature Selection

**Figure 3:**
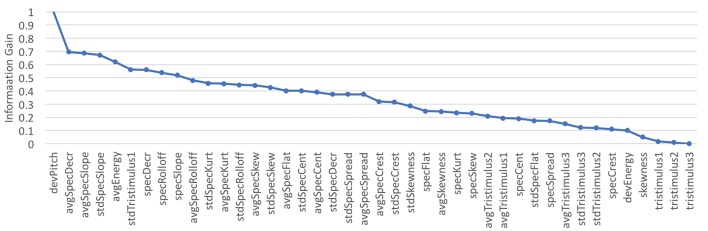
Feature selection: Rankings based on information gain.

**Figure 4:**
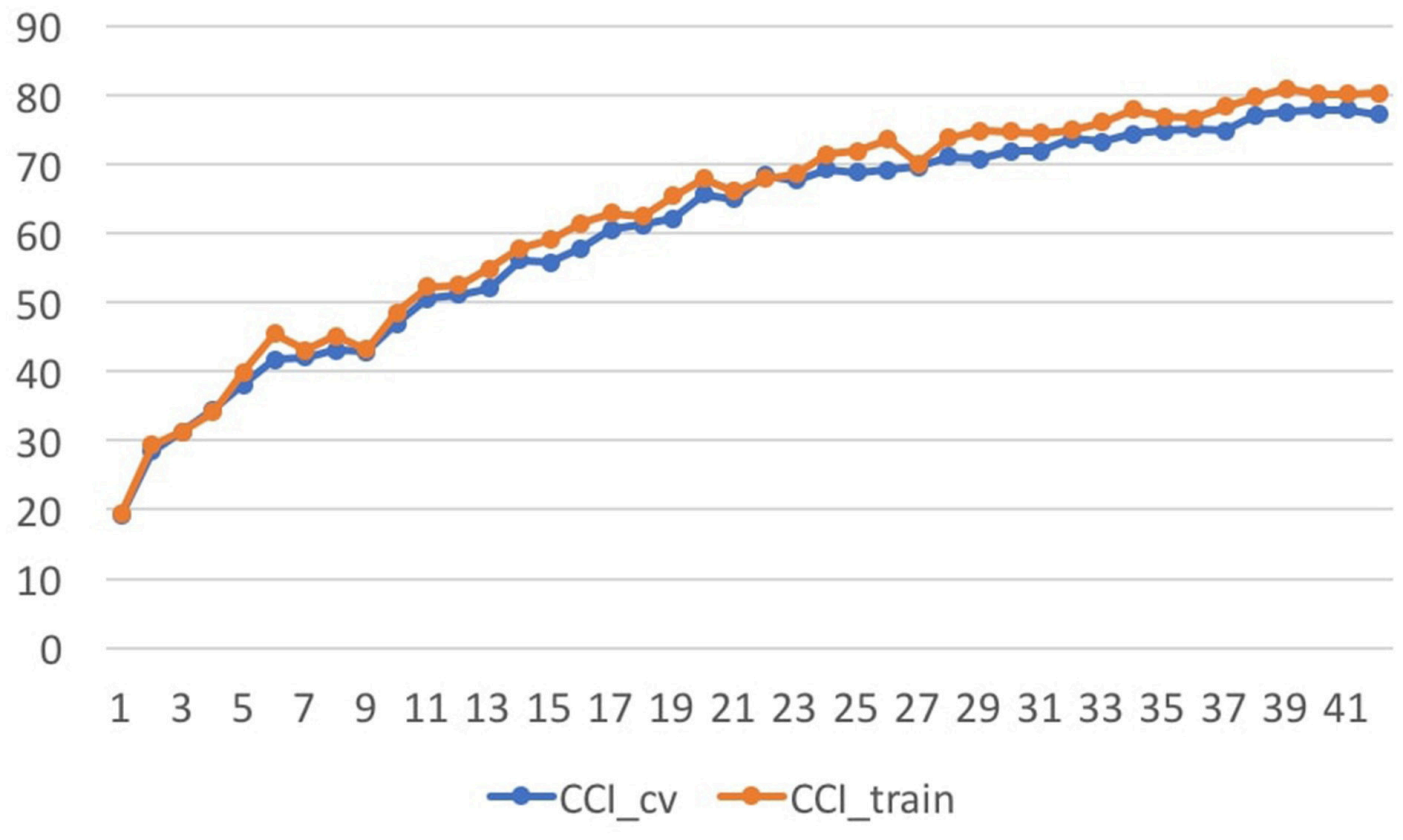
Learning Curves showing the increase of features organized by information gain.

The improvement in accuracy in training the tone quality models over different feature subsets, ranging from 1 feature to all features (incrementally adding features based on their information gain rank) was addressed (see Figure 3). The whole set of recorded examples was considered. Learning curves over the number of instances were obtained to assess the performance of the models over incremental feature subsets (depicted in Figure 4). It can be seen in Figure 4 that the addition of each feature evenly increased the model's accuracy. Therefore, the complete set of features presented in Table 2 was used in the following sections for the modeling stage.

### 4.3. Machine Learning Analysis: Offline

**Figure 5:**
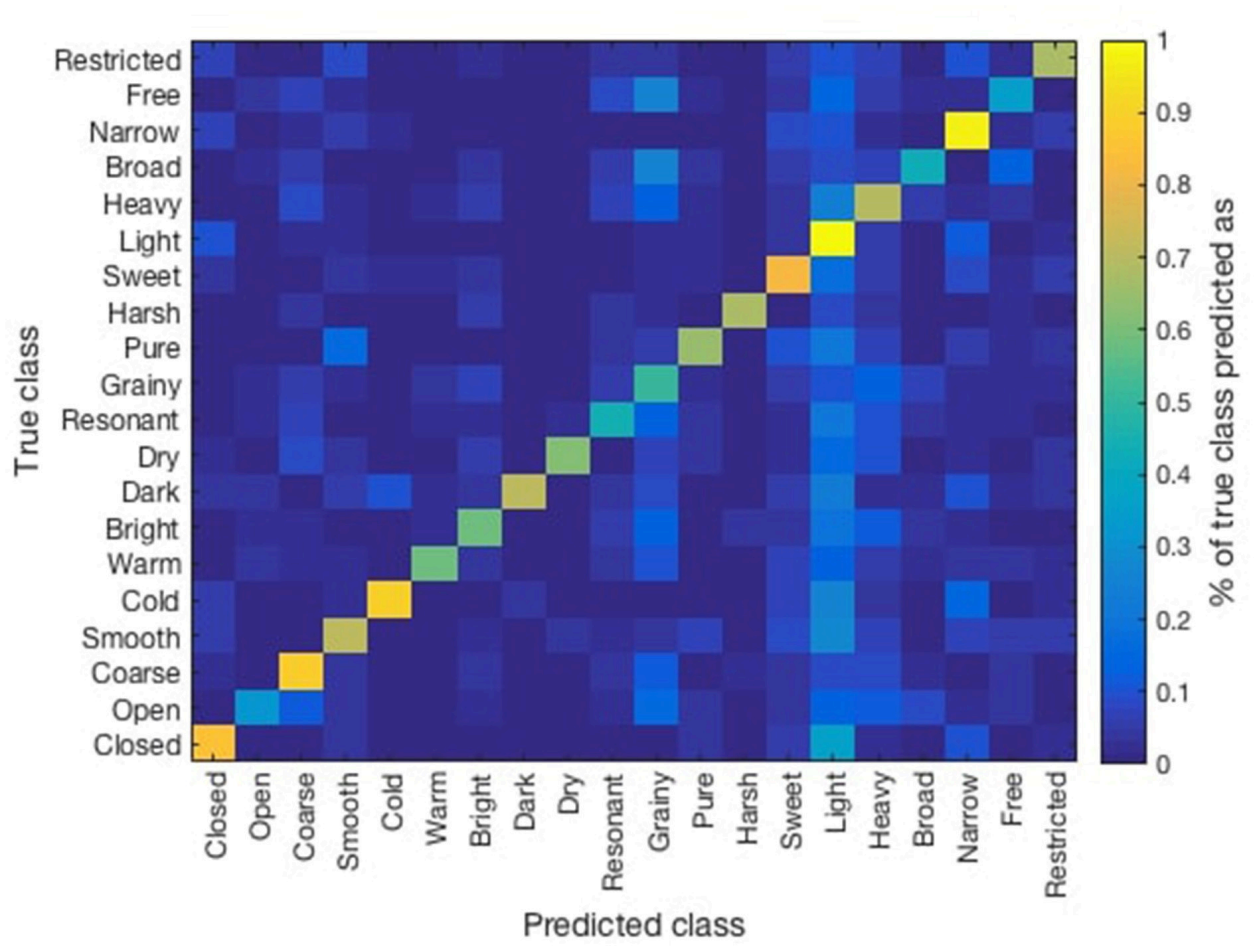
Confusion matrix of the ANN model over the training set.

The results of the different scenarios and sub-groupings taken into consideration are summarized in Table 6 (multi-class), Table 7 (by register), and Table 8 (by position). The evaluation measure used for the models in this section was a Correctly Classified Instances percentage (CCI%) obtained by 10-fold cross validation. The paired *t*-test (*p* < 0.05) showed significant improvement over the baseline in all of the scenarios considered. Similarly, no relevant differences in the accuracy of prediction of the models were observed across the studied scenarios. In Figure 5 we present the confusion matrix for the multi-label classification task for the ANN model. Higher values across the diagonal indicate that the model is able to discriminate the considered classes.

**Table 6:** Multi-class classification accuracies measured as CCI% for Train (T) and 10-fold cross validation (CV).

**Grouping**	**Type**	**Base line**	**Lin. Reg. (T/CV)**	**SVM (T/CV)**	**ANN (T/CV)**
By register	Low	6.41	47.26/52.81	84.02/85.66	88.34/91.6
	Mid	6.59	48.10/53.57	88.54/85.89	87.09/89.57
	High	6.78	47.84/52.08	86.66/86.39	85.03/91.31
By position	Pos. I	6.66	53.27/51.87	89.28/86.34	79.15/77.67
	Pos. V	6.13	49.56/48.51	89.57/86.12	77.88/74.64
By finger	1st.	6.45	48.78/52.37	87.7/85.08	88.67/91.14
	2nd.	6.44	49.76/52.65	86.94/87.50	88.16/90.12
	3rd.	6.76	48.81/51.82	88.9/87.56	86.38/90.65
	4th.	6.64	49.77/52.87	88.81/85.53	87.58/92.91

**Table 7:** Binary classification accuracies measured as CCI% for Train (T) and 10-fold cross validation (CV) for Pitch subgroup.

**Pitch sub-group**	**Class**	**Base line**	**Lin. Reg.**	**SVM**	**ANN**
High	Dark/bright	54.95	89.45 °	92.55 °	98.18 °
	Cold/warm	53.31	90.65 °	92.61 °	98.95 °
	Harsh/sweet	54.59	88.75 °	93.05 °	97.83 °
	Dry/resonant	53.76	87.81 °	92.95 °	98.1 °
	Light/heavy	51.44	89.04 °	92.82 °	97.5 °
	Grainy/pure	50.96	85.24 °	92.79 °	97.3 °
	Coarse/smooth	51.04	87.42 °	94.84 °	98.82 °
	Closed/open	53.48	89.24 °	94.24 °	97.56 °
	Restricted/free	50.96	89.90 °	94.34 °	98.40 °
	Narrow/broad	54.38	86.05 °	94.6 °	97.27 °
Medium	Dark/bright	54.25	89.47 °	92.75 °	97.88 °
	Cold/warm	54.25	90.97 °	94.10 °	97.31 °
	Harsh/sweet	52.62	86.71 °	94.88 °	97.86 °
	Dry/resonant	52.29	88.44 °	92.39 °	97.77 °
	Light/heavy	51.66	87.17 °	93.92 °	97.11 °
	Grainy/pure	54.64	89.94 °	93.48 °	97.71 °
	Coarse/smooth	52.41	85.41 °	93.16 °	97.68 °
	Closed/open	54.77	88.00 °	92.04 °	97.79 °
	Restricted/free	50.41	85.49 °	94.62 °	97.83 °
	Narrow/broad	52.96	87.75 °	94.15 °	97.73 °
Low	Dark/bright	54.62	88.81 °	94.57 °	97.73 °
	Cold/warm	52.14	87.96 °	93.48 °	97.15 °
	Harsh/sweet	51.92	86.25 °	94.12 °	97.85 °
	Dry/resonant	53.31	90.10 °	93.66 °	98.15 °
	Light/heavy	54.95	85.85 °	92.02 °	98.11 °
	Grainy/pure	57.20	87.09 °	93.62 °	98.21 °
	Coarse/smooth	51.52	89.65 °	92.2 °	98.44 °
	Closed/open	53.37	89.95 °	93.15 °	97.18 °
	Restricted/free	53.52	88.82 °	93.72 °	98.78 °
	Narrow/broad	54.52	85.96 °	92.29 °	98.48 °

° *Statistically significant improvement*.

**Table 8:** Binary classification accuracies measured as CCI% for Train (T) and 10-fold cross validation (CV) for Position subgroup.

**Position sub-group**	**Class**	**Base line**	**Lin. Reg**.	**SVM**	**ANN**
First	Closed/open	57.75	88.56 °	86.33 °	95.25 °
	Coarse/smooth	52.62	90.82 °	89.33 °	97.47 °
	Cold/warm	56.30	88.26 °	87.17 °	96.56 °
	Dark/bright	54.10	87.80 °	81.77 °	97.61 °
	Dry/resonant	52.19	82.07 °	80.38 °	92.90 °
	Grainy/pure	54.02	82.81 °	81.06 °	94.69 °
	Harsh/sweet	57.44	86.47 °	82.32 °	97.62 °
	Light/heavy	51.32	88.57 °	85.38 °	93.55 °
	Narrow/broad	54.17	89.21 °	86.49 °	98.25 °
	Restricted/free	54.51	86.14 °	83.82 °	96.41 °
Fifth	Closed/open	58.18	84.79 °	92.97 °	95.02 °
	Coarse/smooth	53.32	86.02 °	94.90 °	97.73 °
	Cold/warm	55.25	80.07 °	91.98 °	95.15 °
	Dark/bright	59.31	83.04 °	91.70 °	97.03 °
	Dry/resonant	52.16	80.68 °	92.96 °	95.13 °
	Grainy/pure	51.51	82.63 °	92.68 °	94.69 °
	Harsh/sweet	53.61	81.46 °	93.37 °	96.80 °
	Light/heavy	55.32	88.99 °	94.20 °	96.18 °
	Narrow/broad	56.44	86.61 °	94.37 °	96.76 °
	Restricted/free	58.71	86.01 °	95.14 °	96.62 °

° *Statistically significant improvement*.

### 4.4. Implementation of a Real-Time Machine Learning Framework

The implementation of the real-time tone quality feedback system is embedded in SkyNote, a general system for automatic assessment of violin performance developed within the scope of the TELMI project (Ramirez et al., 2018). SkyNote computes in real time the frame-based relevant audio descriptors and sends them to the tone quality feedback system through packed lists of OSC messages. The tone-quality system receives these messages and induces and applies the machine learning models. The machine learning component of the system was implemented in C++, based on the OpenFrameworks toolkit (for OSC) and the OpenCV library for machine learning and data processing. It was structured in three different components: data reception/sending, data processing, and machine learning modeling. Data is transmitted through OSC ports between the SkyNote and the tone-quality systems. When received, messages are cataloged into control data, class data, and audio data. Control data is used to control the tone-quality system (e.g., train/run the models, save/load data, etc.). Class data refers to the semantic tone quality label associated to the sound being performed. The label might be one defined by the music experts (default mode), or might be a user-defined label. Finally, audio data refers to the descriptors extracted from the audio performed.

#### 4.4.1. Evaluation

We performed experiments to study if the selected features contained enough information to classify used-defined tonal qualities. We evaluated the system classification accuracy of different (but related) semantic labels, e.g., rich and poor timbre labels. We then compared the accuracy of the user-generated timbre models with that of the “pre-trained” models.

Four professional violinists were asked to record audio examples of rich/poor timbre notes at the four different registers explained in section 3.2, as well as one paired example of a self-defined timbre quality dimension. Each example consisted of a pair of notes, one with the opposite semantic label of the other one which was used as training data. After training users tested the real-time feedback system and recorded again one opposite-label pair of notes for testing. We recorded both the data obtained in real time and the models trained by each subject. We applied Artificial Neural Networks (which produced the best accuracy as seen in section 4.3) for evaluating the obtained models. The average accuracy obtained on the models is presented in Table 9. A similar result in terms of accuracy of the trained models was observed in the offline experiments. We conducted cross validation among performers for the rich/poor tone quality, where each subject tested the system with the trained model of the other three violinists. To avoid bias based on the violin quality, performers used the violin corresponding to the performer who trained the model. In this case, the accuracy of the models tended to decrease (see Table 10). This might be due to the fact that performers use different performance resources to produce the same tonal quality.

**Table 9:** Binary classification accuracies measured as CCI% for the real-time framework tests.

	**Class**	**Base line**	**ANN**
Subject 1	Rich/poor	52.62	98.32 °
Subject 2	Rich/poor	54.63	98.41 °
Subject 3	Rich/poor	51.45	98.23 °
Subject 4	Rich/poor	54.62	97.38 °
Subject 1	Light/heavy	51.30	98.40 °
Subject 2	Bad/good	53.96	97.30 °
Subject 3	Thin/full	51.02	97.84 °
Subject 4	Light/heavy	53.14	98.73 °

° *Statistically significant improvement*.

**Table 10:** Binary classification accuracies measured as CCI% for cross validation (CV) among performers and models.

		**Models trained by**			
		**Subject 1**	**Subject 2**	**Subject 3**	**Subject 4**
Test note by	Subject 1	92.08	58.33	63.29	55.26
	Subject 2	88.97•	92.44	64.79	62.83
	Subject 3	55.96	57.61	92.67	57.62
	Subject 4	56.99	56.03	56.23	92.34

• *Accuracy obtained by subject 2 on model by subject 1 after several trials*.

## 5. Discussion

We have presented a machine learning approach for the automatic assessment of the quality of tone in violin performance. We have obtained a list of 10 opposite semantic tonal dimensions provided by violin experts. We obtained recordings of each of the tonal dimensions performed by a professional violinist and performed a listening test of the provided dimensions using an online survey. The semantic associations of listeners over the defined tonal dimensions were studied based on inter-user and inter-dimension correlations. Spectral low-level descriptors were extracted from the recording examples to later train machine learning models. An offline machine learning approach was performed to investigate the accuracy obtained with three different learning schemes, as well as across several performance scenarios (different fingering, register, and position). A subset of features was selected for a real-time approach, where the system extracted in real-time the aforementioned set of descriptors and provided real-time feedback on the quality of the proposed tonal dimensions. The system is able to be re-trained with user-defined sound examples and semantic labels. An evaluation of the accuracy of the user-trained models was performed in which it was observed that the selected set of features contained enough information to correctly classify different intended performed tonal qualities.

In general, participants in the perceptual study pointed out that the differences across the different tone examples were so subtle that it was difficult to remember previous samples, which may explain the lack of consensus among participants. However, computers have perfect memory and are able to extract features characterizing the acoustic features of the audio samples which seem to be informative for differentiating the samples. Furthermore, in the cross validation tests among performers, some participants managed to tune the performance resources in order to match the tonal quality model trained by a second violinist. The majority of existing studies have looked at very different tones either synthetically generated or across instruments. However, given the fact that in this study we are considering several variables simultaneously (several audio descriptors, several performers, several listeners) it might be the case that, in the real world, the differences among intended performance tonal qualities are often quite subtle.

Most of the systems reviewed in section 2 approach the correctness of a performance by assessing its timing and pitch accuracy, whereas the system presented in this paper deals with the quality of sound produced by the performer. In this sense, the work by Romani Picas et al. (2015) is the most related existent system, which assesses timbre richness and timbre stability as part of the dimensions used for sound quality. However, this system is based on static/predefined models of sound quality trained with recordings made under specific conditions (i.e. specific room acoustics, microphones, and musical instruments). The specificity of the data used to train the system produces models which lack generality and are inaccurate when audio capture conditions vary.

Initial versions of our system were also trained using recordings made under specific conditions (e.g., acoustic conditions, quality of the instrument, level of the performer, etc.) and, as the system proposed by Romani Picas et al. (2015), failed to generalize. In addition to this, due to the subjectiveness of the adjectives musicians use to describe music timbre (e.g., cold, warm, dark, bright) timbre description varied across performers. In order to solve these issues, the system proposed in this paper allows the possibility of the user to train the sound quality models. Thus, each user trains and uses the system using the same audio capture conditions and has control over the semantics of the tone labels he/she defines.

The similarity of some of the considered tonal dimensions might be better addressed on a comparative type test, where users can provide a rating of a particular tone quality based on the possibility of listening to several audio samples. Similarly, a large scale study with violin performers could be performed to obtain the semantic labels following a similar methodology used by Saitis et al. (2017). For the tone survey a closer study of the variation of RMS and pitch could be addressed to confirm some of the claims/comments provided by listeners regarding associations among some perceived tonal qualities and the level of vibrato and loudness.

The evaluation of the system and its user-defined approach, including implications for practice in music education contexts, will be addressed in future work.

## Author Contributions

SG, RR, GW, AP, and AW contributed conception and design of the study. SG, AP, RR, GW, and AO were involved the data acquisition. SG, GW, and AO designed and programed the platform for the survey data acquisition. SG performed the statistical analysis. OM led the main software implementation framework. IN and SG performed the main programming tasks. SG wrote the first draft of the manuscript. All authors contributed to manuscript revision.

### Conflict of Interest Statement

The handling editor is currently editing co-organizing a Research Topic with one of the authors AW, and confirms the absence of any other collaboration. The authors declare that the research was conducted in the absence of any commercial or financial relationships that could be construed as a potential conflict of interest.

## Footnotes

^1^www.yousician.com

^2^www.smartmusic.com

^3^Data deposited at https://github.com/chechojazz/TelmiToneStudyData
